# Inflammation and necrosis syndrome is associated with alterations in blood and metabolism in pigs

**DOI:** 10.1186/s12917-021-03107-1

**Published:** 2022-01-19

**Authors:** Frederik Loewenstein, Sabrina Becker, Josef Kuehling, Hansjörg Schrade, Mirjam Lechner, Robert Ringseis, Klaus Eder, Andreas Moritz, Gerald Reiner

**Affiliations:** 1 Department of Veterinary Clinical Sciences, Clinic for Swine, Justus-Liebig-University, Frankfurter Strasse 112, 35392 Giessen, Germany; 2 LSZ Boxberg, Seehöfer Str. 50, 97944 Boxberg, Germany; 3 UEG Hohenlohe, Am Wasen 20, 91567 Herrieden, Germany; 4 Institute of Animal Nutrition and Nutrition Physiology, Justus Liebig University Giessen, Heinrich-Buff-Ring 26-32, 35392 Giessen, Germany; 5 Department of Veterinary Clinical Sciences, Clinic for Small Animals, Justus-Liebig-University, Frankfurter Strasse, 35392 Giessen, Germany

**Keywords:** Swine, Tail biting, Metabolism, Haemostasis, Blood cell counts

## Abstract

**Background:**

Swine inflammation and necrosis syndrome (SINS) can lead to significant clinical alterations at tail, ears, claws and other parts of the body in suckling piglets, weaners and fatteners. Clinical findings are associated with vasculitis, intima proliferation and thrombosis. The syndrome can be found in newborns, indicating a primarily endogenous aetiology. It has been hypothesized that SINS is triggered by gut-derived microbial-associated molecular patterns, causing derangements in liver metabolism and activity of peripheral white blood cells involving inflammation and blood haemostasis. In order to characterize these metabolic derangements of SINS for the first time, red and white blood counts, parameters of blood haemostasis, serum metabolites and acute phase proteins in the serum were analysed in 360 piglets, weaners and fatteners, each with significantly different SINS scores.

**Results:**

SINS scores and haematological/clinical chemical parameters were significantly associated (*P* < 0.05), especially in weaners and fatteners. Higher degrees of clinical SINS were associated with increased numbers of monocytes and neutrophils. Blood coagulation was altered in weaners and a thrombocytopenia was found in fatteners. Additionally, acute phase proteins, especially C-reactive protein and fibrinogen were increased in serum. Serum metabolites and serum liver enzymes were slightly altered. Aspartate transaminase levels overall exceeded physiological limit and increased in parallel with SINS scores in fatteners.

**Conclusion:**

Clinical inflammation and necrosis at tail, ears, claws and other parts of the body were significantly associated with haematology and serum clinical chemistry, especially in weaners and fatteners. The involvement of inflammatory cells, blood coagulation, acute phase proteins and certain serum metabolites support the inflammatory-necrotising character of the syndrome and provide starting points for further studies to decipher its exact pathogenesis. The low to moderate variations seem less suitable for diagnostic use.

**Supplementary Information:**

The online version contains supplementary material available at 10.1186/s12917-021-03107-1.

## Background

Swine inflammation and necrosis syndrome (SINS) can lead to significant clinical signs in swine at different ages [1, 2], on tail, ears, heels, claw coronary bands, teats, umbilicus, vulva and face. Clinical findings are in accordance with histopathological signs of inflammation, e.g. vasculitis, intima proliferation and thrombosis of the vessels, even in animals with macroscopically intact epidermis [2, 3]. Such associations were reported already in newborn piglets. Due to the involvement of macrophages and lymphocytes on the histopathological alterations in the newborns no later than 2 h after birth, technopathies or biting could be ruled out as the cause for these lesions [3].

Taken together, these facts indicate a primarily endogenous aetiology of SINS. A causative role of circulatory disorders in the development of inflammation and necrosis at the tails of suckling piglets was discussed in earlier studies [4–6]. It is supported by the histopathological evidence of vasculitis and thrombosis at the tail base proximal to the lesions [3], and by a sudden drop in the tail bases’ temperature in affected piglets, measured by thermal imaging camera [7].

Inflammation and necrosis can seriously impair animal welfare because they are painful and can be substrate for microbial infection [8, 9], and injuries to the skin, tail and ears have been used to characterize animal welfare in pigs. Thus, SINS could be relevant for the assessment of impaired animal welfare in pigs.

We hypothesize that SINS is triggered by local processes at the blood vessels of the acra. Necroses at the tail, ears or coronary bands were observed in piglets suckled by sows treated with deoxynivalenone (DON) and lipopolysaccharides (LPS) [10–12]. A comprehensive synthesis of how systemic inflammation can synergistically be caused by microbiota, blood-gut barrier, immune activation, mycotoxins, nutritional status, feed composition, housing environment, hygiene and even psychological stress was recently published by Nordgreen et al. [13]. LPS and other microbial components are generally termed microbial-associated molecular patterns (MAMPs). MAMPs can be translocated from the healthy intestine to the liver via the portal vein (for overview see [13]). Liver-resident macrophages (Kupffer cells) and other intrahepatic immune cells largely prevent the entry of MAMPs into the systemic circulation [14, 15]. If the gut barrier is disrupted (“leaky”) and the liver is subjected to high levels of MAMPs, they are recognized not only by hepatic immune cells but also by the abundant MAMP recognition receptors of hepatic parenchymal cells. Activation of receptors for MAMPs, such as Toll-like receptors (TLR), nucleotide-binding domain and leucine-rich-repeat-containing proteins (NLR) and others, by MAMP stimulates various inflammatory and stress-signalling pathways and results in hepatic inflammation and an impaired functionality of the organ [16]. Subsequently, MAMPs enter the circulatory system and lead to systemic inflammation and alterations of the blood haemostasis [17, 18]. Following this context, we hypothesized that SINS is indirectly triggered by gut-derived MAMPs (for an overview see [13]) entering the liver and the circulatory system. This would cause derangements in liver metabolism [19–22], i.e., a switch to an acute phase and inflammatory response, and activate peripheral white blood cells involved in inflammation and blood haemostasis [17, 18]. Evidence that SINS is associated with hepatic inflammation and extensive changes in the hepatic transcriptome in suckling piglets has recently been provided [23] and led to the expectation of metabolic derangement in affected piglets. In order to characterize these expected metabolic derangements of SINS piglets for the first time, red and white blood counts, parameters of blood haemostasis, serum metabolites and acute phase proteins in the serum were analysed in piglets with significant differences in SINS scores. The piglets were examined in three age groups, as suckling piglets, weaners and fatteners. On the one hand, such data could provide clues to the cause and development of the disease, and on the other hand, new diagnostic approaches could be expected.

## Results

### Clinical SINS scores and alterations of different body parts

The examined animals weighted 1.69 ± 0.26 kg at birth, 1.76 ± 0.30 kg 3 days after birth, 7.19 ± 1.22 kg 3 weeks after birth, 10.06 ± 1.61 kg 11 days after weaning and 118.4 ± 6.8 kg at slaughter (data not shown). Fifty-one percent of pigs were female.

**Fig. 1:**
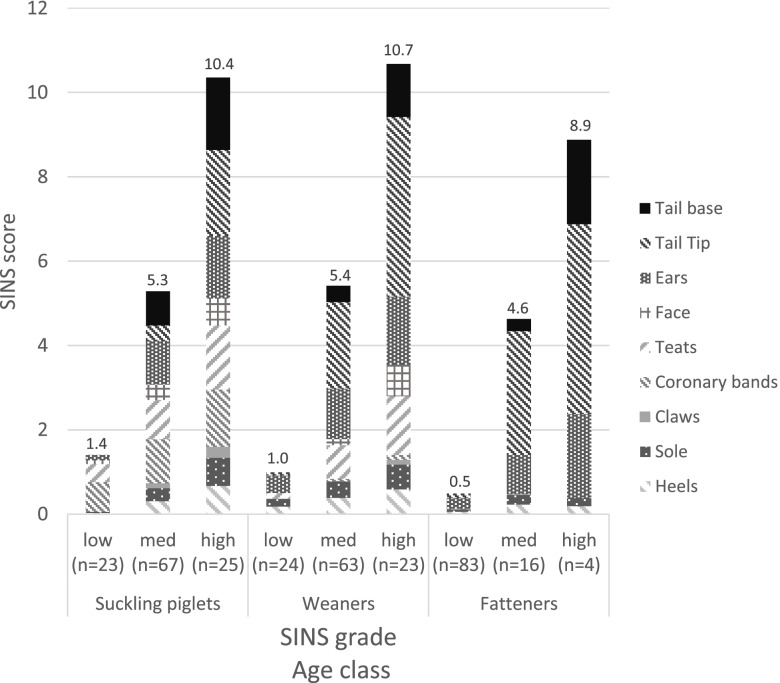
SINS score and scores of the individual body parts by age class and SINS gradesgroup (low, medium and high). The figure shows cumulative means. Total SINS scores are given above columns. Numbers of cases (n) are given in brackets

After division of the pigs into three groups of ascending SINS grade (Fig. 1; low, med, high), the SINS scores of the low group (the 20% of pigs with the lowest scores) ranged from 0 to 2.25, those of the medium group (60% of the pigs) ranged from 2.5 to 8, and those of the high group (the 20% of the pigs with the highest scores) ranged from 8.25 to 14.5. At all ages, the SINS scores as well as all individual organ scores differed significantly among the three groups, except for teats, face, and coronary bands in fatteners (for details see Additional file 1).The distribution of SINS grades in suckling piglets and weaners were similar. In the fatteners, there were only few animals with medium or high SINS scores (see respective numbers in Fig. 1).

Regardless of the age group, there were no signs at the tail base in the group with low SINS scores. Also, on the rest of the tail, scabs, exudation and slight necrosis occurred only in a maximum of 5.6% of the fatteners and 4.2% of the weaners. In contrast, the base of the tail and the tail of > 70 to 100% of the weaners and fatteners in the group with high SINS grades were affected by bristle loss, swelling and exudation (Fig. 1, Additional Table 1). The significant correlations between prevalence of signs and SINS score were also seen in the ears, face and teats (Fig. 1, Additional Table 2). Differences between SINS groups were less pronounced at the claws (Fig. 1, Additional Table 3). Alterations also occurred in the group with low SINS grades with regard to the coronary bands, claw walls, soles and to a higher degree to the heels. Nevertheless, the SINS groups also differed significantly from each other for the characteristics of the claw scores. Taken together, the clinical findings of the three groups differed significantly and substantially in all body parts examined.

### Haematology and clinical chemistry

Blood count and metabolic parameters were still largely unaffected by SINS score in the suckling piglets (Tables 1, 2, 3, 4, 5). The only notable abnormalities associated with higher SINS scores were high C-reactive protein (CRP) levels (Table 3) and lower total protein and globulin levels (Table 4).

**Table 1:** White blood count according to SINS grades

Parameter	Age group	SINS low	SINS med	SINS high	r	P
WBC (10^9^/L)	SP	6.95 ± 1.89	8.06 ± 3.85	7.85 ± 2.55	0.059	n.s.
	WP	18.32 ± 6.08	19.27 ± 5.25	18.08 ± 4.4	− 0.037	n.s.
	FA	19.78 ± 3.82	21.35 ± 6.94	17.6 ± 6.49	− 0.056	n.s.
LYM (10^9^/L)	SP	2.31 ± 0.93	2.53 ± 1.31	2.4 ± 1.03	−0.021	n.s.
	WP	8.12 ± 2.82	7.44 ± 2.82	6.24 ± 2.7	**−0.205**	**0.031**
	FA	11.66 ± 3.36	11.49 ± 4.32	11.81 ± 4.34	0.023	n.s.
LYM% (%)	SP	33.85 ± 12.09	32.64 ± 12.65	31.35 ± 10.46	− 0.092	n.s.
	WP	46.81 ± 12.8	39.85 ± 14.2	35.31 ± 15.03	**−0.263**	**0.005**
	FA	58.68 ± 14.66	52.45 ± 15.3	67.18 ± 0.19	0.100	n.s.
MON (10^9^/L)	SP	0.59 ± 0.18	0.54 ± 0.23	0.58 ± 0.2	0.027	n.s.
	WP	0.28 ± 0.58	0.6 ± 0.84	0.94 ± 0.85	**0.254**	**0.007**
	FA	0.41 ± 0.68	0.81 ± 1.13	0.11 ± 0.05	−0.178	n.s.
MON (%)	SP	8.43 ± 0.94	7.15 ± 2.57	7.7 ± 2.24	−0.068	n.s.
	WP	1.34 ± 2.38	3 ± 3.88	5.16 ± 4.41	**0.291**	**0.002**
	FA	2.06 ± 3.28	3.39 ± 4.4	0.65 ± 0.13	−0.146	n.s.
NEU (10^9^/L)	SP	4.05 ± 1.46	4.99 ± 3.66	4.87 ± 2.11	0.092	n.s.
	WP	9.91 ± 4.61	11.23 ± 4.7	10.9 ± 3.93	0.054	n.s.
	FA	7.71 ± 2.83	9.04 ± 4.33	5.67 ± 2.1	− 0.145	n.s.
NEU (%)	SP	57.71 ± 12.03	60.11 ± 13.55	61.05 ± 10.81	0.103	n.s.
	WP	51.85 ± 11.14	57.14 ± 11.56	60.21 ± 11.09	**0.256**	**0.007**
	FA	38.61 ± 11.17	41.16 ± 12.22	32.2 ± 0.18	− 0.149	n.s.

*Hb* haemoglobin, *HCT* haematocrit, *LYM* lymphocytes, *MCH* mean corpuscular haemoglobin, *MCHC* mean corpuscular haemoglobin concentration, *MCV* mean corpuscular volume, *MON* monocytes, *NEU* neutrophils, *RBC* red blood cell, *RDW* red cell distribution, *WBC* white blood cell, *SP* suckling piglets, *WP* weaners, *FA* fatteners, *r* correlation coefficient for the respective parameters with the SINS scores, *P* significance of r, *n.s.* not significant

**Table 2:** Platelets and coagulation profiles according to SINS grades

Parameter	Age group	SINS low	SINS med	SINS high	r	P
PLT (10^9^/L)	SP	296.9 ± 90.9	280.6 ± 118.4	307.4 ± 73.7	0.066	n.s.
	WP	414.3 ± 208.4	447.2 ± 166.0	407.5 ± 136.4	− 0.091	n.s.
	FA	285.5 ± 87.2	235.3 ± 96.9	177.8 ± 142.8	**− 0.319**	**0.001**
PLT (%)	SP	0.33 ± 0.1	0.32 ± 0.14	0.35 ± 0.1	0.057	n.s.
	WP	0.41 ± 0.2	0.44 ± 0.16	0.41 ± 0.13	−0.075	n.s.
	FA	0.28 ± 0.08	0.22 ± 0.09	0.19 ± 0.17	**−0.248**	**0.011**
MPV (fL)	SP	11.13 ± 0.66	11.2 ± 1.05	11.48 ± 1.04	0.089	n.s.
	WP	9.83 ± 0.89	9.97 ± 1.03	10.23 ± 0.87	0.150	n.s.
	FA	9.74 ± 0.95	9.39 ± 0.64	9.78 ± 2.02	0.170	n.s.
PDW (%)	SP	41.99 ± 1.16	41.12 ± 4.26	42.08 ± 1.87	−0.024	n.s.
	WP	38.13 ± 6.55	39.61 ± 2.22	40.27 ± 1.58	0.179	n.s.
	FA	39.17 ± 3.96	39.51 ± 1.57	39.28 ± 4.97	0.113	n.s.
PT (sec)	SP	14.77 ± 0.87	14.96 ± 1.37	14.66 ± 1.05	−0.019	n.s.
	WP	12.7 ± 0.71	13.22 ± 0.73	13.94 ± 1.81	**0.375**	**< 0.001**
	FA	12.81 ± 0.65	12.74 ± 0.62	12.7 ± 0.71	−0.029	n.s.
APTT (sec)	SP	17.55 ± 1.8	17.37 ± 1.84	17.57 ± 2.02	−0.021	n.s.
	WP	14.9 ± 1.76	15.24 ± 2.19	16.68 ± 2.35	**0.298**	**0.003**
	FA	11.23 ± 3.12	12.7 ± 1.19	10.9 ± 0.28	0.100	n.s.
PTINR	SP	1.17 ± 0.09	1.19 ± 0.14	1.18 ± 0.09	0.058	n.s.
	WP	0.96 ± 0.07	1.01 ± 0.07	1.09 ± 0.18	**0.375**	**0.004**
	FA	0.95 ± 0.17	0.97 ± 0.06	0.96 ± 0.07	−0.026	n.s.
AT-III (%)	SP	48.43 ± 8.56	53.35 ± 12.68	52.76 ± 9.13	0.128	n.s.
	WP	93.55 ± 10	94.27 ± 9.76	102 ± 7.28	**0.288**	**0.004**
	FA	105 ± 8.11	106.89 ± 6.47	115 ± 7.55	0.052	n.s.
D-dimers (uGu/mL)	SP	0.3 ± 0.09	0.54 ± 0.92	0.21 ± 0.15	**−0.256**	**0.007**
	WP	0.14 ± 0.05	0.15 ± 0.08	0.12 ± 0.07	−0.122	n.s.
	FA	0.28 ± 0.25	0.24 ± 0.14	0.29 ± 0.25	−0.094	n.s.

*APTT* activated partial thromboplastine time, *AT-III* antithrombin III activity, *MPV* mean platelet volume, *PDW* platelet distribution width, *PLT* platelets, *PT* prothrombin time, *PTINR* prothrombin time – international normalized ratio, *SP* suckling piglets, *WP* weaners, *FA* fatteners, *r* correlation coefficient for the respective parameters with the SINS scores, *P* significance of r, *n.s.* not significant

**Table 3:** Serum acute phase proteins according to SINS grades

Parameter	Age group	SINS low	SINS med	SINS high	r	P
Haptoglobin (g/L)	SP	82.8 ± 37.9	69.7 ± 61.4	51.9 ± 15.9	**−0.308**	**0.001**
	WP	88.6 ± 72.2	107.0 ± 90.5	125.5 ± 181.3	0.042	n.s.
	FA	416.0 ± 331.8	370.4 ± 230.7	491.8 ± 399.6	0.051	n.s.
CRP (nmol/L)	SP	13.3 ± 17.3	21.1 ± 34.9	30.1 ± 34.1	**0.258**	**0.005**
	WP	103.8 ± 55.4	109.3 ± 56.9	112.9 ± 56.6	0.033	n.s.
	FA	106.2 ± 56.6	135.6 ± 45.4	150 ± 0	**0.229**	**0.020**
Fibrinogen (g/L)	SP	1.05 ± 0.19	1.13 ± 0.44	1.12 ± 0.21	0.103	n.s.
	WP	1.66 ± 0.44	1.71 ± 0.53	1.77 ± 0.93	− 0.053	n.s.
	FA	1.87 ± 0.63	2.93 ± 0.89	3.33 ± 0.24	**0.453**	**< 0.001**

*CRP* C-reactive protein, *SP* suckling piglets, *WP* weaners, *FA* fatteners, *r* correlation coefficient for the respective parameters with the SINS scores, *P* significance of r, *n.s.* not significant

**Table 4:** Serum metabolites according to SINS grades

Parameter	Age group	SINS low	SINS med	SINS high	r	P
Urea (g/dL)	SP	34.05 ± 21.26	34.65 ± 24.98	35.31 ± 18.97	− 0.032	n.s.
	WP	12.67 ± 7.38	9.25 ± 5.83	6.73 ± 4.92	**− 0.327**	**< 0.001**
	FA	28.71 ± 7.21	30.69 ± 7.21	39.94 ± 2.40	**0.262**	**0.008**
Creatinine (g/dL)	SP	0.56 ± 0.10	0.59 ± 0.21	0.58 ± 0.08	0.103	n.s.
	WP	0.97 ± 0.20	0.96 ± 0.20	0.89 ± 0.18	− 0.085	n.s.
	FA	1.86 ± 0.31	1.81 ± 0.13	1.92 ± 0.31	0.080	n.s.
Total Protein (g/dL)	SP	6.36 ± 1.37	5.83 ± 1.18	5.70 ± 0.83	**− 0.200**	**0.032**
	WP	4.43 ± 0.55	4.41 ± 0.74	4.24 ± 0.76	**− 0.194**	**0.043**
	FA	7.98 ± 1.24	7.27 ± 0.52	7.55 ± 0.52	−0.135	n.s.
Albumin (g/dL)	SP	1.18 ± 0.19	1.22 ± 0.40	1.16 ± 0.17	−0.065	n.s.
	WP	2.69 ± 0.35	2.72 ± 0.389	2.80 ± 0.51	0.133	n.s.
	FA	4.57 ± 0.64	4.26 ± 0.26	4.46 ± 0.40	−0.099	n.s.
Globulin (g/dL)	SP	5.18 ± 1.28	4.62 ± 1.04	4.54 ± 0.75	**−0.220**	**0.019**
	WP	1.74 ± 0.36	1.69 ± 0.53	1.45 ± 0.41	**−0.336**	**< 0.001**
	FA	3.41 ± 0.77	3.02 ± 0.61	3.10 ± 0.36	**−0.209**	**0.034**
Glucose (mg/dL)	SP	120.7 ± 25.0	117.6 ± 24.1	111.5 ± 20.0	−0.118	n.s.
	WP	166.6 ± 38.6	154.0 ± 29.7	157.5 ± 36.8	−0.108	n.s.
	FA	127.6 ± 45.6	133.7 ± 30.1	105.4 ± 26.3	0.093	n.s.
Bilirubin (mg/dL)	SP	0.37 ± 0.23	0.38 ± 0.21	0.29 ± 0.24	− 0.112	n.s.
	WP	0.12 ± 0.08	0.09 ± 0.05	0.06 ± 0.004	**− 0.349**	**< 0.001**
	FA	0.24 ± 0.15	0.22 ± 0.10	0.30 ± 0.12	0.034	n.s.
Cholesterin (mg/dL)	SP	106.3 ± 35.2	102.1 ± 30.2	105.2 ± 24.7	0.015	n.s.
	WP	65.3 ± 14.3	64.2 ± 11.6	68.4 ± 11.2	0.136	n.s.
	FA	103.6 ± 15.9	109.4 ± 12.4	121.0 ± 5.0	0.167	n.s.
Triglycerides (mg/dL)	SP	165.4 ± 112	129.5 ± 96.3	172.4 ± 112	0.065	n.s.
	WP	41.1 ± 11.4	38.5 ± 16.6	40.3 ± 14.9	0.014	n.s.
	FA	64.8 ± 21.9	70.9 ± 24.5	113.8 ± 42.9	**0.307**	**0.002**

*SP* suckling piglets, *WP* weaners, *FA* fatteners, *r* correlation coefficient for the respective parameters with the SINS scores, *P* significance of r, *n.s.* not significant

**Table 5:** Serum enzymes according to SINS grades

Parameter	Age group	SINS low	SINS med	SINS high	r	P
AP (U/L)	SP	2591 ± 987	1970 ± 799	2087 ± 995	−0.187	0.054
	WP	191 ± 51	177 ± 62	194 ± 69	−0.008	n.s.
	FA	175 ± 62	167 ± 62	209 ± 47	−0.046	n.s.
ALT (U/L)	SP	47.1 ± 14.5	42.8 ± 9.7	45.4 ± 30.2	−0.173	n.s.
	WP	29.6 ± 7.7	31.5 ± 8.4	34.4 ± 11.3	**0.189**	**0.048**
	FA	56.6 ± 12.1	53.2 ± 9.54	62.3 ± 6.3	−0.001	n.s.
GLDH (U/L)	SP	6.61 ± 14.71	3.31 ± 7.41	2.12 ± 2.05	0.033	n.s.
	WP	1.25 ± 1.07	1.84 ± 1.13	2.52 ± 1.88	**0.330**	**< 0.001**
	FA	1.62 ± 1.19	1.4 ± 0.97	2 ± 2	−0.023	n.s.
CK (U/L)	SP	257 ± 186	336 ± 856	159 ± 71	**−0.245**	**0.008**
	WP	2034 ± 2250	3044 ± 3686	4187 ± 7855	0.090	n.s.
	FA	4051 ± 3434	2469 ± 939	2961 ± 1176	−0.058	n.s.
AST (U/L)	SP	11.65 ± 6.64	15.75 ± 15.78	12.36 ± 8.91	−0.043	n.s.
	WP	57.88 ± 31.71	65 ± 51.62	61.3 ± 36.69	−0.011	n.s.
	FA	37.11 ± 25.73	58.4 ± 24.49	62 ± 17.4	**0.243**	**0.013**

*AP* alkaline phosphatase, *ALT* alanine aminotransferase, *GLDH* glutamate dehydrogenase, *CK* creatin kinase, *AST* aspartate aminotransferase, *SP* suckling piglets, *WP* weaners, *FA* fatteners, *r* correlation coefficient for the respective parameters with the SINS scores, *P* significance of r, *n.s.* not significant

**Fig. 2:**
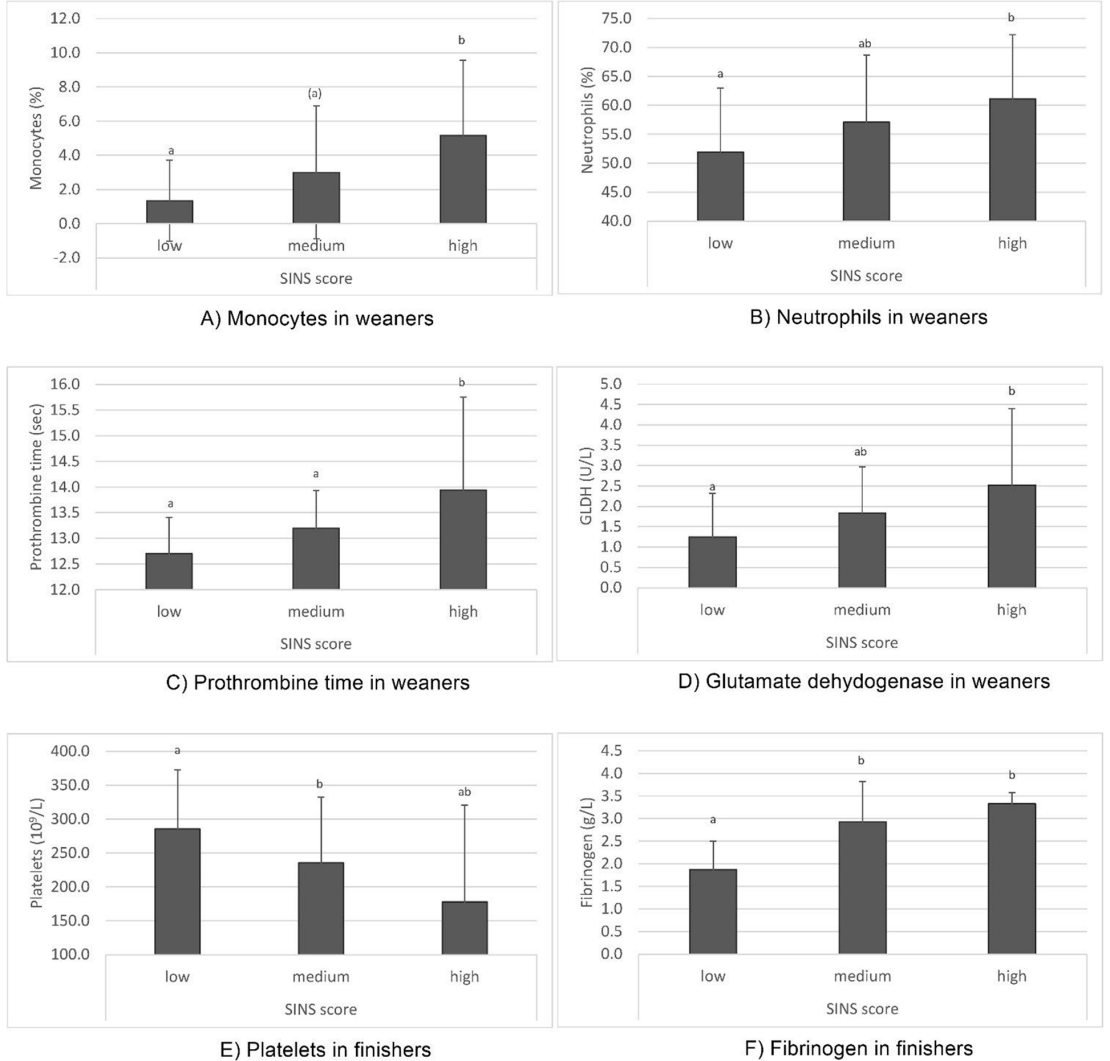
Associations of SINS scores with different haematological and clinical chemical parameters in weaners and finishers Bars represent means ± standard deviations. Groups with the same letter are statistically not significant (*P* > 0.05) or, if the letter is in brackets (*P* > 0.07). Significances are Bonferroni corrected

Most associations between SINS score and metabolic parameters were found in the weaners age group. Higher SINS scores were associated with higher numbers and proportions of monocytes (Fig. 2A) and neutrophils (Fig. 2B), and with lower proportions of lymphocytes (Table 1). Increasing SINS scores were also correlated with prolonged prothrombin time (PT; Fig. 2C), prolonged activated thromboplastin time (aPTT), increased prothrombin time – international normalized ratio (PTINR) and increased anti-thrombin III activity (Table 2). Associations of SINS scores with CRP or fibrinogen were not visible at that age class (Table 3).

Serum levels of urea, total protein, bilirubin and globulin decreased with rising SINS scores (Table 4). Serum enzymes characterizing liver cell integrity were in the physiological range for alanine aminotransferase (ALT; ≤ 68 U/L) and glutamate dehydrogenase (GLDH; ≤ 4 U/L; Fig. 2D), as expected, but aspartate aminotransferase (AST) levels were above the physiological range of 35 U/L (Table 5). Additionally, serum ALT and GLDH were significantly and positively correlated with SINS scores.

Despite some differences between weaners and fatteners, the associations between SINS score and metabolic parameters were present even at the highest age group. Associations with monocyte or neutrophil counts could no longer be demonstrated. However, with increasing SINS scores, there was a decrease in platelet counts (Fig. 2E) and proportions (Table 2). At the same time, fibrinogen (Fig. 2F) and CRP levels were significantly higher in pigs with higher SINS scores (Table 3). Total bilirubin exceeded the threshold for pathologically elevated values (≤ 0.25) in the group with the highest SINS scores (Table 4). AST levels overall exceeded the physiological limit (≤ 35) and increased in parallel with SINS scores (Table 5 Variation between low and high SINS groups was small in red blood cell parameters (see Additional Table 4) and serum electrolyte levels (see Additional Table 5) and showed no remarkable association with SINS scores.

## Discussion

The present study showed significant associations between the SINS score and haematological and clinical-chemical parameters. According to the classification, the degrees of inflammation of the individual body regions increased significantly in pigs from the low to the high SINS group. At the same time, however, significant age differences were observed. In the suckling piglets, strong clinical signs of inflammation were present in the areas of the tail base, face, teats, coronary bands, and heels, together with pronounced wall bulging. All these signs were reduced with increasing age. These body regions are particularly sensitive in newborn piglets [3]. In contrast, alterations in the tail, apart from the base, the ears, bleeding into the claw wall and detachment of the sole and heels did not yet play a role in suckling piglets and only occurred in weaners and fatteners. These findings agree well with the results of Reiner et al. [2]. Alterations in the tail seem to be well associated with inflammation of the tail base [2]. Ring constrictions and hemorrhages of the tail occurred almost exclusively in weaners. Detachment of the sole and wall was likely due to an interaction with floor, to which pigs were exposed for longer periods of time as they aged, although an influence of SINS was also demonstrated for these parameters. It is reasonable to expect that alterations that increase with age may be due to an interaction between pre-damage by SINS and external mechanical irritation.

Associations between signs of SINS and haematological/clinical chemical parameters were not yet pronounced at the 3rd day of life (suckling piglets). Only elevated CRP levels were indicative for an inflammatory status in higher SINS grades at that age. Increased CRP levels in severely SINS-affected suckling piglets are in line with results from our recent transcriptomic study, in which an upregulation of acute phase proteins and proinflammatory genes was found in the liver of suckling piglets with high-grade clinical signs of SINS compared with piglets without significant signs of SINS [23]. This indicates that SINS-affected piglets experience an inflammatory condition which is not only restricted to the liver but obviously also becomes systemic. Low globulin levels at high SINS grades may indicate that a poorer supply of maternal antibodies could promote the development of SINS. However, the fraction of globulins contains not only gamma globulins and proportions can be extremely variable. We did not differentiate between the fractions. Although CRP can be a hint on an inflammatory status of the liver in suckling piglets, the hypothesiszed hepatic dysfunction does not yet appear to be fully developed at this age.

Associations with SINS scores became particularly evident around the 40th day of life (weaners). Monocytes and neutrophils now appeared in greater numbers, while numbers of lymphocytes declined. Matching the observations of hemorrhages in the tail and claw wall area, delayed blood clotting was evident. At the same time, serum metabolites were decreased and liver enzymes were elevated. These parameters in weaners are indicative for inflammation, thrombosis and coagulation problems.

When assessing the values of the fatteners, however, it must be taken into account that only relatively few animals with moderate or high SINS scores were present. At around 5.5 months of age, the tightness of the associations declined slightly compared to weaners, but was still significant. Elevated fibrinogen and CRP values still indicated inflammatory processes. Significantly decreased platelet numbers are likely to be a consequence of the coagulation disorders observed in the weaners. The already pathologically elevated AST values in the weaners under SINS were still evident in the fatteners. It can be assumed that the hypothesized dysfunction of the liver in accordance with higher SINS grades is not expressed until weaning age and is not completely improving until the end of the fattening period.

There were no indications of acute or chronic swine diseases despite regular and comprehensive, clinical and laboratory examinations, e.g. for Porcine Reproductive and Respiratory Syndrome Virus (PRRSV), Porcine Circovirus Type 2 (PCV2), *Actinobacillus pleuropneumoniae*, *Glaeserella parasuis*; Enzootic pneumonia, diarrhoeal diseases.

Associations between SINS and features of blood coagulation were particularly striking in the present study. Interactions between inflammatory processes and blood coagulation are well understood [17, 18, 24–26]. Severe coagulopathies in systemic inflammation can occur in the form of disseminated intravascular coagulation (DIC) [27, 28]. Microvascular thrombosis [29, 30] with associated ischemic necrosis [31] has also been described in association with DIC. Vasculitis and thrombosis were shown to be the histopathological background of clinical SINS [3], including the same animals examined in the present study [2].

In the present study, the white blood count of affected piglets was characterized by a significant increase in monocytes and neutrophils at the expense of lymphocytes. Associations between white blood cells and SINS score occurred in weaners only. Somewhat later, in the fatteners, a decrease in platelet numbers was observed. Monocytes play a central role in the recognition of tissue damage and in the initiation of appropriate countermeasures (for review see [18]). These cells can be activated by cytokines, endotoxin (LPS) and other damage- or pathogen-associated molecular patterns (DAMPs, PAMPs) via pathogen recognition receptors such as TLRs. In addition to other cytokines, the tissue factor (TF) is also centrally involved in the start of a complex defense cascade [18]. It activates blood coagulation and thrombus formation, leukocytes and various defense systems (e.g. the complement system). Weaners with high SINS in particular, showed increased PT, PTINR and APTT values. Somewhat later, in the fatteners, increasing SINS scores were associated with a reduction of platelet numbers, reaching the state of thrombocytopenia in the highest SINS score group. This raises the suspicion of a consumption coagulopathy. It is matched by the fact that APTT in weaners was prolonged beyond the normal range in the groups with medium and high SINS scores.

Although absolute values did not reach pathological levels (> 120%), an increase in antithrombin III, which was clearly pronounced in the weaners, might be an indication of an acute inflammatory reaction, together with the clinical signs of SINS in different body parts. The increased monocyte and neutrophil levels also indicate inflammatory [32–34] and necrotic processes [35–37], and they were significantly associated with SINS scores in weaners. Neutrophils promote immigration of monocytes from the blood vessels at the site of the lesion. They are also involved in tissue regeneration [38–40]. In addition, increasing monocyte levels may be associated with ischemic infarcts [41–43]. The elevated levels of CRP, which were already detected in suckling piglets and again, to a particular extent in fatteners, support the assumption of a vascular disease, as elevated CRP levels were also described in artherosclerosis [44]. These findings agree well with the clinical findings of SINS and with histopathological defects detected by Reiner et al. [2] and Kühling et al. [3]. Acute phase proteins are part of the systemic acute phase response and components of the innate immune system. The concentration of acute phase proteins in the plasma is altered in animals subjected to challenges such as infection, inflammation, trauma or stress [45]. Their synthesis takes place mainly in the liver under the stimulus of the pro-inflammatory cytokines IL-1β, IL-6 and TNF-α [46]. A complex regulatory network linking the cytokine response elicited by the pathogen and the unique regulation of each acute phase protein gene by cytokines leads to an extremely variable response [47]. Among the acute phase proteins, CRP was more informative than haptoglobin (HP) in the present study, and there were some significant differences between the age groups. Such differences and effects are not surprising. In some studies, CRP was the only clearly increased acute phase protein in serum [48], sometimes with delay [49], while HP showed best results in other studies [47, 50]. CRP plays a significant role in the clearance of infectious agents and damaged cells, through its ability to bind phosphocholine [51]. Thus, from the clinically and histopathologically demonstrated inflammation and necrosis in SINS, including vasculitis and thrombosis, and from the hypothesis that SINS is indirectly triggered by gut-derived MAMPs, an increase in CRP was expected. It remains, however, unclear, why suckling piglets with highest SINS scores had the lowest levels of HP.

One of the clearest associations in the age group of the fatteners was found between SINS score and fibrinogen levels. Some pigs with highest SINS score exceeded physiological levels (3.9 g/L). The liver protein fibrinogen is an acute phase protein and a clotting factor. It stabilizes platelets, but also increases plasma viscosity, aggregation of red blood cells and platelets, and thus may also contribute to the risk of thrombosis [52, 53]. The mechanisms underlying this increase require further study but this could be another link between acute phase and circulatory disturbances supporting the development of inflammation and necrosis in SINS.

A possible indication for a reduced synthesis capacity of the liver, as expected by Ringseis et al. [23] was found in the APTT. This indication was supported by decreasing urea values with increasing SINS scores in weaners. These urea values were below the physiological range (20–50 g/dL) and could generally be associated with a decreased food intake in the days after weaning. This aspect might have been more pronounced in SINS piglets, as it is well known, how inflammation can lead to centrally reduced appetite and a reduction of food intake [13]. Urea levels can be also indicators of body protein catabolism, but there were no consequences for the piglets’ performance, as weight gain and fattening performance were not traceably affected by SINS in the studied piglets [2]. The kidney could also in parts be responsible for varying urea levels. The role of the kidney remains unclear, however, as creatinine values in the weaners were within the physiological range (0.45–1.5 mg/dL). Pathologically high creatinine values were reached in fatteners only, regardless of the animals’ SINS score. Lower levels of serum total protein, globulines and bilirubin, together with the increase in liver enzyme levels with growing SINS scores, might also be indicative for an association between SINS and liver metabolism. Associations between SINS and red cell counts in fatteners might be partly due to haemo-concentration, but a possible explanation is missing.

The results of the present study do not suggest that the metabolic variations were causative for SINS or vice versa. There were many indications that the clinical manifestations of SINS and the alterations in blood and metabolism occurred synchronously, instead, possibly triggered by the same factors. Describing the exact aetiology and pathogenesis should be the subject of future research, because such information could help to better combat the syndrome and to improve pig welfare by reducing inflammation and necrosis and the associated haematological and clinical chemical alterations.

## Conclusion

In conclusion, there were clear indications of associations between clinical SINS grade on the one hand and altered blood coagulation with thrombocytopenia, low-grade monocytosis and neutrophilia, an increase in the acute phase proteins CRP and fibrinogen and a low-grade influence on liver metabolism on the other hand. The absolute deviations of the examined parameters, however, were in a low range. Only a few parameters were elevated or reduced beyond their normal values. This result is in line with expectations because the inflammatory alterations in SINS, although leading to a distinct clinical pathology do not cause and are not accompanied by life-threatening conditions, such as e.g. endotoxin shock. Therefore, no high specificity of haematological and clinical-chemical symptomatology was observed that could be used diagnostically.

## Methods

All parts of the experiment including the evaluation were performed in a double-blinded manner. This is not a randomized study. Clinical, hematological, and clinical chemistry examinations of all animals were performed in a blinded fashion. After completion of all examinations, associations between clinical parameters and hematological/clinical chemistry parameters were examined.

### Animals and experimental design

The animal experiment was carried out in the conventional stables of the State Institution for Swine Breeding (Landesanstalt für Schweinezucht, LSZ) Baden-Wurttemburg in Boxberg, Germany. The experiments were performed with offspring of 40 Baden-Wurttemburg Genetics hybrid sows artificially inseminated with Pietrain semen. Sows were not randomly selected. Instead, two groups of sows, one with the least and one with the most severe alterations in claws and teats, were selected and the experiment was carried out in two successive repetitions with different husbandry conditions. Because sows’ quality and husbandry are not the topic of the present paper, more information about sow selection and husbandry can be found in Reiner et al. [2].

A total of 360 pigs were used in the experiment. Nine offspring per sow from the 40 sows were successively examined in three age groups: Three piglets per sow as suckling piglets on the third day of life, three others per sow as weanlings 11 days after weaning and the remaining three piglets per sow as fattening pigs during slaughter. All non-slaughtered pigs were euthanized immediately after the clinical examination and sampled for further examination [2].

### Clinical scoring

Piglets were clinically scored for SINS on the third day of life as described by Reiner et al. [2]. Due to time constraints and to minimize the animal load, clinical signs were recorded using a digital camera (Lumix, Panasonic Corporation) according to a standardized scheme for later detailed evaluation of the images (Windows Media Player, Version 12, Microsoft GmbH, Germany). The tail base and tip, ears, teats and navel, coronary bands, wall horn, balls and soles of the feet along with the face were all initially assessed individually. The following clinical characteristics were considered and scored with 0 if the sign was not visible or 1 if the sign was visible. The tail base was screened for the presence of bristles (0 = present; 1 = absent), swelling of the tail base (0/1), redness of the tail base (0/1), exudation (0/1) and clinical signs of necrosis (0/1). The tail, including the whole tail except the tail base was scored for swelling (0/1), scab formation (0/1), rhagades (0/1), exudation (0/1), necrosis (0/1), bleeding (0/1) and ring lacings (0/1). Ears were scored for the presence of bristles (0 = present; 1 = absent), ear vein combustion (0/1) and necrosis of the ears (0/1). The face was scored for lid oedema (0/1) and nasal oedema (0/1). Teats were scored for scab formation (0/1), swelling (0/1), redness (0/1), necrosis (0/1) and venous combustion (0/1). Each claw was individually scored for redness of coronary band (0/1), exudation of coronary band (0/1) and necrosis of coronary band (0/1), for wall bulging (0/1) and wall bleeding (0/1), for sole redness (0/1) and detachment of sole from heel (0/1), and for swelling (0/1), redness (0/1) and detachment of heels (0/1). In the end, mean scores of the eight claws, resp. 4 ft were used for the calculation of the organ scores. For the other body parts, all detailed scores were added to produce organ scores. All organ scores were unweighted summed up for the SINS-Score. SINS-Scores between 0 and 31 were theoretically possible.

### Sample collection

After the clinical scoring, the piglets were sampled by collection of 4.5 ml serum (Monovette Serum 4.5 ml, Sarstedt AG), 2.5 ml citrate whole blood (S-Monovette® 2.9 ml 9NC, Citrate, Sarstedt AG) and 2.5 ml EDTA whole blood (S-Monovette® 2.7 ml K3E, EDTA, Sarstedt AG) from the cranial vena cava using a 1.2 × 4.0 mm cannula (Agani, Terumo Corporation).

### Sample processing and examination

The collected EDTA whole blood was processed approx. 4–6 h after collection. First, 1000 μl were pipetted into EDTA sample tubes (Sarstedt AG, Germany) using a volumetric pipette (Eppendorf AG, Germany). The haematological examination was performed with a VetScan HM5 (Abaxis Europe GmbH) using a VetScan HM5 Reagent Pack (Abaxis Europe GmbH). The examination profile animal species pig was selected for a 3-fold differentiation. The parameters determined included the red blood count with the individual values RBC, HGB, HCT, MCV, MCH, MCHC, RDW, PLT, PCT, MPV and PDW, and the white blood count with WBC, LYM, MON, NEU, LYM in %, MON in % and NEU in %.

The collected citrated whole blood was centrifuged approx. 4–6 h after collection at 1500 rpm for 30 min with a Labofuge 400 (Heraeus, Thermo Fisher Scientific Inc.). The plasma supernatant was pipetted into microcentrifuge tubes (Sarstedt AG) using a volumetric pipette (Eppendorf AG) and stored at − 21 °C for the investigation of the coagulation profile.

The coagulation parameters were determined with a STA Compact (Stago Deutschland GmbH). The profile included values for fibrinogen, prothrombin time (PT), activated partial thromboplastin time (APTT), prothrombin time – international normalized ratio (PTINR), antithrombin III (AT-III) and D-dimers.

The collected serum-blood samples were centrifuged approx. 4–6 h after collection at 3000 rpm for 5 min with a Labofuge 400 (Heraeus, Thermo Fisher Scientific Inc.). The serum was pipetted with a volumetric pipette (Eppendorf AG) into microcentrifuge tubes (Sarstedt AG) and stored at − 21 °C for further investigations.

For the CRP (C-reactive protein) analysis, the samples were sent to Biocheck GmbH in Leipzig. There, the analysis was carried out using an ELISA (Phase Porcine CRP Assay Kit, Tridelta Development Ltd.) according to the manufacturer’s instructions.

The clinical blood chemistry parameters were determined using an ABX Pentra 400 (Horiba Europe GmbH). Urea, creatinine, total protein, albumin, globulin, glucose, bilirubin, cholesterol, triglycerides, alkaline phosphatase (AP), alanine aminotransferase (ALT), glutamate dehydrogenase (GLDH), aspartate aminotransferase (AST), creatine kinase (CK) and haptoglobin were measured. The electrolyte values sodium, chloride, potassium, calcium, phosphate and magnesium were determined using the Nova Electrolyte Analyzer (Nova Biomedical GmbH).

### Statistical analysis

All data were analysed with the Program Packagge IBM-SPSS, V.26, Munich, Germany.

For the present study, all individuals were classified according to their SINS score. The 20% individuals with the lowest SINS scores were assigned to the SINS low group, the 20% individuals with the highest SINS scores to the SINS high group, and the remaining individuals (60%) to the SINS med (medium) group. Clinical, haematological and clinical-chemical characteristics were compared between these groups. Because several animals had the same SINS score, the exact proportions of 20% could not be kept. Because the fatteners hadsignificantly lower SINS scores, the group with low SINS scores admitted considerably more animals than the group with high SINS scores in this age group. Means ± standard deviations of the three groups were given for all parameters (0/1) and scores. Frequencies of alterations in the parameters were calculated by chi^2^-Test, Scores were analysed by univariate analysis of variance. Correlation coefficients were calculated for all parameters with the SINS-Score and significances for these correlations were given as the distribution independent Spearman’s correlation coefficient. An α-level of < 0.003 was considered as statistically significant for clinical parameters, after Bonferroni adjustment (original: α < 0.1). Haematological and clinical chemical parameters were considered as statistical significant at α < 0.1, respectively at α < 0.002 after Bonferroni correction. *P* values in Tables are the original values (no Bonferroni correction).

## Supplementary Information

**Additional file 1.** Containing Additional Tables 1 to 5.

## Data Availability

Data and materials are available from the authors on reasonable request.

## Abbreviations

ALT: Alanine aminotransferase
AP: Alkaline phosphatase
APTT: Activated partial thromboplastine time
AST: Aspartate aminotransferase
AT-III: Antithrombin III activity
CK: Creatin kinase
CRP: C-reactive protein
GLDH: Glutamate dehydrogenase
Hb: Haemoglobin
HCT: Haematocrit
LYM: Lymphocytes
MCV: Mean corpuscular volume
MCH: Mean corpuscular haemoglobin
MCHC: Mean corpuscular haemoglobin concentration
MON: Monocytes
MPV: Mean platelet volume
NEU: Neutrophils
P: Significance
PDW: Platelet distribution width
PLT: Platelets
PCT: Percentage of platelets in white cell count
PT: Prothrombin time
PTIN: Prothrombin time – international normalized ratio
r: Correlation
RBC: Red blood cell
RDW: Red cell distribution
SINS: Swine inflammation and necrosis syndrome
WBC: White blood cell
