# Supplementary material for: Inflammation and necrosis syndrome is associated with alterations in blood and metabolism in pigs
Source: BMC Vet Res. 2022 Jan 19;18:50. doi: 10.1186/s12917-021-03107-1 (PMC8767723; doi:10.1186/s12917-021-03107-1)
Supplement: Supplementary file 1 — Additional file 1. Containing Additional Tables 1 to 5. [file 12917_2021_3107_MOESM1_ESM.docx]

**Additional File 1**

**Additional Table 1:** Score and prevalence of clinical signs at the tail base and tail according to SINS grades

| Tail Base | Age group | | SINS low | | SINS med | | SINS high | | r | P |  |
| --- | --- | --- | --- | --- | --- | --- | --- | --- | --- | --- | --- |
| Score | SP | | 0 ± 0 | | 0.81 ± 0.91 | | 1.72 ± 1.14 | | 0.640 | <0.001 |  |
|  | WP | | 0 ± 0 | | 0.39 ± 0.7 | | 1.26 ± 0.86 | | 0.666 | <0.001 |  |
|  | FA | | 0 ± 0 | | 0.29 ± 0.61 | | 2 ± 0 | | 0.392 | <0.001 |  |
| No bristles (%) | SP | | 0 | | 40.3 | | 72.0 | | 0.524 | <0.001 |  |
|  | WP | | 0 | | 18.8 | | 43.5 | | 0.423 | <0.001 |  |
|  | FA | | 0 | | 14.3 | | 100.0 | | 0.349 | <0.001 |  |
| Swelling (%) | SP | | 0 | | 35.8 | | 76.0 | | 0.586 | <0.001 |  |
|  | WP | | 0 | | 17.2 | | 73.9 | | 0.648 | <0.001 |  |
|  | FA | | 0 | | 7.1 | | 100.0 | | 0.305 | 0.002 |  |
| Redness (%) | SP | | 0 | | 3.0 | | 12.0 | | 0.188 | 0.044 |  |
|  | WP | | 0 | | 1.6 | | 8.7 | | 0.232 | 0.015 |  |
|  | FA | | 0 | | 0.0 | | 0.0 | | 0.000 | n.s. |  |
| Exudation (%) | SP | | 0 | | 1.5 | | 8.0 | | 0.186 | 0.047 |  |
|  | WP | | 0 | | 1.6 | | 0.0 | | 0.039 | n.s. |  |
|  | FA | | 0 | | 7.1 | | 0.0 | | 0.170 | n.s. |  |
| Necrosis (%) | SP | | 0 | | 0.0 | | 4.0 | | 0.155 | n.s. |  |
|  | WP | | 0 | | 0.0 | | 0.0 | | 0.000 | n.s. |  |
|  | FA | | 0 | | 0.0 | | 0.0 | | 0.000 | n.s. |  |
| Tail tip | |  | |  | |  | |  |  |  | |
| Score | | SP | | 0 ± 0 | | 0.36 ± 0.81 | | 2.04 ± 1.51 | 0.677 | <0.001 | |
|  | | WP | | 0.08 ± 0.41 | | 2.05 ± 1.48 | | 4.26 ± 1.01 | 0.848 | <0.001 | |
|  | | FA | | 0.11 ± 0.42 | | 2.93 ± 1.44 | | 4.5 ± 0.71 | 0.729 | <0.001 | |
| Swelling (%) | | SP | | 0 | | 13.4 | | 48.0 | 0.443 | <0.001 | |
|  | | WP | | 0 | | 20.3 | | 87.0 | 0.697 | <0.001 | |
|  | | FA | | 0 | | 64.3 | | 100.0 | 0.558 | <0.001 | |
| Scab (%) | | SP | | 0 | | 9.0 | | 44.0 | 0.498 | <0.001 | |
|  | | WP | | 4.2 | | 68.8 | | 100.0 | 0.709 | <0.001 | |
|  | | FA | | 5.7 | | 85.7 | | 100.0 | 0.678 | <0.001 | |
| Rhagades (%) | | SP | | 0 | | 1.5 | | 40.0 | 0.450 | <0.001 | |
|  | | WP | | 0 | | 14.1 | | 65.2 | 0.611 | <0.001 | |
|  | | FA | | 0 | | 14.3 | | 50.0 | 0.302 | 0.002 | |
| Exudation (%) | | SP | | 0 | | 9.0 | | 60.0 | 0.560 | <0.001 | |
|  | | WP | | 4.2 | | 65.6 | | 95.7 | 0.713 | <0.001 | |
|  | | FA | | 3.4 | | 78.6 | | 100.0 | 0.624 | <0.001 | |
| Necrosis (%) | | SP | | 0 | | 3.0 | | 12.0 | 0.245 | 0.008 | |
|  | | WP | | 0 | | 35.9 | | 78.3 | 0.564 | <0.001 | |
|  | | FA | | 2.3 | | 50.0 | | 100.0 | 0.539 | <0.001 | |
| Bleeding (%) | | SP | | 0 | | 3.0 | | 0.0 | 0.053 | n.s. | |
|  | | WP | | 0 | | 17.2 | | 26.1 | 0.290 | 0.002 | |
|  | | FA | | 0 | | 0.0 | | 0.0 | 0.000 | n.s. | |
| Ring lacings (%) | | SP | | 0 | | 0.0 | | 0.0 | 0.000 | n.s. | |
|  | | WP | | 0 | | 1.6 | | 4.3 | 0.131 | n.s. | |
|  | | FA | | 0 | | 0.0 | | 0.0 | 0.000 | n.s. | |

SP: suckling piglets; WP; weaners; FA: fatteners; r: correlation coefficient for the respective parameters with the SINS scores; P: significance of r; n.s.: not significant.

**Additional Table 2:** Score and alterations at the ears, face and teats according to SINS grades

| Ears | Age group | | SINS low | | SINS med | SINS high | | | r | P | |
| --- | --- | --- | --- | --- | --- | --- | --- | --- | --- | --- | --- |
| Score | SP | | 0.13 ± 0.46 | | 1.04 ± 0.79 | 1.48 ± 0.65 | | | 0.560 | <0.001 | |
|  | WP | | 0.42 ± 0.58 | | 1.2 ± 0.67 | 1.61 ± 0.58 | | | 0.558 | <0.001 | |
|  | FA | | 0.25 ± 0.49 | | 0.93 ± 0.73 | 2 ± 0 | | | 0.691 | <0.001 | |
| No bristles (%) | SP | | 4.3 | | 62.7 | 64.0 | | | 0.415 | <0.001 | |
|  | WP | | 4.2 | | 46.9 | 69.6 | | | 0.501 | <0.001 | |
|  | FA | | 2.3 | | 21.4 | 100.0 | | | 0.405 | <0.001 | |
| Venous combustion (%) | SP | | 8.7 | | 41.8 | 84.0 | | | 0.508 | <0.001 | |
|  | WP | | 37.5 | | 73.4 | 91.3 | | | 0.377 | <0.001 | |
|  | FA | | 23 | | 71.4 | 100.0 | | | 0.677 | <0.001 | |
| Face |  | |  |  | | |  |  | | |  |
| Score | SP | | 0.09 ± 0.29 | 0.37 ± 0.52 | | | 0.64 ± 0.64 | 0.435 | | | <0.001 |
|  | WP | | 0 ± 0 | 0.14 ± 0.35 | | | 0.74 ± 0.75 | 0.525 | | | <0.001 |
|  | FA | | 0 ± 0 | 0 ± 0 | | | 0 ± 0 |  | | |  |
| Lid edema (%) | SP | | 8.7 | 35.8 | | | 56.0 | 0.422 | | | <0.001 |
|  | WP | | 0 | 14.1 | | | 56.5 | 0.519 | | | <0.001 |
|  | FA | | 0 | 0.0 | | | 0.0 |  | | | n.s. |
| Nose edema (%) | SP | | 0 | 1.5 | | | 8.0 | 0.229 | | | 0.014 |
|  | WP | | 0 | 0.0 | | | 17.4 | 0.297 | | | 0.002 |
|  | FA | | 0 | 0.0 | | | 0.0 |  | | | n.s. |
| Teats |  |  | | |  |  | | |  |  | |
| Score | SP | 0.43 ± 0.51 | | | 0.93 ± 0.84 | 1.52 ± 0.96 | | | 0.423 | <0.001 | |
|  | WP | 0.13 ± 0.45 | | | 0.8 ± 0.78 | 1.39 ± 1.03 | | | 0.460 | <0.001 | |
|  | FA | 0.01 ± 0.11 | | | 0 ± 0 | 0 ± 0 | | | 0.096 | n.s. | |
| Scab (%) | SP | 30.4 | | | 43.3 | 48.0 | | | 0.089 | n.s. | |
|  | WP | 12.5 | | | 15.6 | 39.1 | | | 0.283 | 0.003 | |
|  | FA | 1.1 | | | 0.0 | 0.0 | | | 0.096 | n.s. | |
| Swelling (%) | SP | 17.4 | | | 29.9 | 60.0 | | | 0.341 | <0.001 | |
|  | WP | 4.2 | | | 32.8 | 34.8 | | | 0.234 | 0.014 | |
|  | FA | 1.1 | | | 0.0 | 0.0 | | | 0.096 | n.s. | |
| Redness (%) | SP | 4.3 | | | 7.5 | 16.0 | | | 0.139 | n.s. | |
|  | WP | 4.2 | | | 31.3 | 73.9 | | | 0.461 | <0.001 | |
|  | FA | 0 | | | 0.0 | 0.0 | | | 0.000 | n.s. | |
| Necrosis (%) | SP | 0 | | | 3.0 | 12.0 | | | 0.216 | 0.02 | |
|  | WP | 0 | | | 0.0 | 4.3 | | | 0.161 | n.s. | |
|  | FA | 0 | | | 0.0 | 0.0 | | | 0.000 | n.s. | |
| Venous combustion (%) | SP | 21.7 | | | 52.2 | 64.0 | | | 0.284 | 0.002 | |
|  | WP | 4.2 | | | 15.6 | 26.1 | | | 0.199 | 0.038 | |
|  | FA | 0 | | | 0.0 | 0.0 | | | 0.000 | n.s. | |

SP: suckling piglets; WP; weaners; FA: fatteners; r: correlation coefficient for the respective parameters with the SINS scores; P: significance of r; n.s.: not significant.

**Additional Table 3:** Score and alterations at the claws according to SINS grades

| Coronary bands | | Age group | | SINS low | | | SINS med | | | SINS high | | | r | | | P | |  |
| --- | --- | --- | --- | --- | --- | --- | --- | --- | --- | --- | --- | --- | --- | --- | --- | --- | --- | --- |
| Score | | SP | | 0.7 ± 0.51 | | | 1.04 ± 0.61 | | | 1.36 ± 0.63 | | | 0.361 | | | <0.001 | |  |
|  | | WP | | 0 ± 0 | | | 0.05 ± 0.14 | | | 0.12 ± 0.21 | | | 0.308 | | | 0.001 | |  |
|  | | FA | | 0 ± 0 | | | 0 ± 0 | | | 0 ± 0 | | |  | | | n.s. | |  |
| Redness (%) | | SP | | 39.1 | | | 56.7 | | | 80.0 | | | 0.277 | | | 0.003 | |  |
|  | | WP | | 0 | | | 7.8 | | | 30.4 | | | 0.328 | | | <0.001 | |  |
|  | | FA | | 0 | | | 0.0 | | | 0.0 | | |  | | | n.s. | |  |
| Exudation (%) | | SP | | 87 | | | 92.5 | | | 100.0 | | | 0.219 | | | 0.018 | |  |
|  | | WP | | 0 | | | 9.4 | | | 13.0 | | | 0.171 | | | n.s. | |  |
|  | | FA | | 0 | | | 0.0 | | | 0.0 | | |  | | | n.s. | |  |
| Necrosis (%) | | SP | | 8.7 | | | 28.4 | | | 32.0 | | | 0.228 | | | 0.014 | |  |
|  | | WP | | 0 | | | 1.6 | | | 4.3 | | | 0.059 | | | n.s. | |  |
|  | | FA | | 0 | | | 0.0 | | | 0.0 | | |  | | | n.s. | |  |
| Claw wall | |  |  | | |  | | |  | | |  | | |  | | |  |
| Score | | SP | 0 ± 0 | | | 0.12 ± 0.19 | | | 0.26 ± 0.23 | | | 0.516 | | | <0.001 | | |  |
|  | | WP | 0.01 ± 0.05 | | | 0.01 ± 0.05 | | | 0.11 ± 0.21 | | | 0.201 | | | 0.036 | | |  |
|  | | FA | 0 ± 0 | | | 0.02 ± 0.07 | | | 0 ± 0 | | | 0.161 | | | n.s. | | |  |
| Bulging (%) | | SP | 0 | | | 34.3 | | | 68.0 | | | 0.517 | | | <0.001 | | |  |
|  | | WP | 4.2 | | | 4.5 | | | 26.1 | | | 0.194 | | | 0.042 | | |  |
|  | | FA | 0 | | | 7.1 | | | 0.0 | | | 0.161 | | | n.s. | | |  |
| Bleeding (%) | | SP | 0 | | | 6.0 | | | 8.0 | | | 0.105 | | | n.s. | | |  |
|  | | WP | 20.8 | | | 30.3 | | | 52.2 | | | 0.271 | | | 0.004 | | |  |
|  | | FA | 4.6 | | | 14.3 | | | 50.0 | | | 0.242 | | | 0.014 | | |  |
| Soles |  | | | |  | | |  | | |  | | |  | | |  | |
| Score | SP | | | | 0.03 ± 0.08 | | | 0.31 ± 0.37 | | | 0.67 ± 0.38 | | | 0.626 | | | <0.001 | |
|  | WP | | | | 0.18 ± 0.26 | | | 0.39 ± 0.34 | | | 0.59 ± 0.41 | | | 0.417 | | | <0.001 | |
|  | FA | | | | 0.06 ± 0.14 | | | 0.23 ± 0.27 | | | 0.19 ± 0.27 | | | 0.472 | | | <0.001 | |
| Redness (%) | SP | | | | 17.4 | | | 56.7 | | | 88.0 | | | 0.518 | | | <0.001 | |
|  | WP | | | | 54.2 | | | 74.2 | | | 82.6 | | | 0.241 | | | 0.011 | |
|  | FA | | | | 11.5 | | | 21.4 | | | 50.0 | | | 0.280 | | | 0.004 | |
| Detachment | SP | | | | 0 | | | 6.0 | | | 12.0 | | | 0.151 | | | n.s. | |
| (%) | WP | | | | 12.5 | | | 31.8 | | | 34.8 | | | 0.124 | | | n.s. | |
|  | FA | | | | 16.1 | | | 64.3 | | | 50.0 | | | 0.455 | | | <0.001 | |
| Heels |  | | | |  | | |  | | |  | | |  | | |  | |
| Score | SP | | | | 0.03 ± 0.08 | | | 0.31 ± 0.37 | | | 0.67 ± 0.38 | | | 0.626 | | | <0.001 | |
|  | WP | | | | 0.18 ± 0.26 | | | 0.39 ± 0.34 | | | 0.59 ± 0.41 | | | 0.417 | | | <0.001 | |
|  | FA | | | | 0.06 ± 0.14 | | | 0.23 ± 0.27 | | | 0.19 ± 0.27 | | | 0.472 | | | <0.001 | |
| Swelling (%) | SP | | | | 100 | | | 100.0 | | | 100.0 | | |  | | | n.s. | |
|  | WP | | | | 95.8 | | | 89.4 | | | 100.0 | | | 0.184 | | | n.s. | |
|  | FA | | | | 56.3 | | | 85.7 | | | 100.0 | | | 0.231 | | | 0.019 | |
| Redness (%) | SP | | | | 95.7 | | | 94.0 | | | 88.0 | | | -0.077 | | | n.s. | |
|  | WP | | | | 41.7 | | | 65.2 | | | 69.6 | | | 0.194 | | | 0.043 | |
|  | FA | | | | 5.7 | | | 35.7 | | | 50.0 | | | 0.277 | | | 0.005 | |
| Detachment | SP | | | | 0 | | | 0.0 | | | 0.0 | | |  | | | n.s. | |
| (%) | WP | | | | 25 | | | 39.4 | | | 30.4 | | | 0.071 | | | n.s. | |
|  | FA | | | | 16.1 | | | 21.4 | | | 100.0 | | | 0.184 | | | n.s. | |

SP: suckling piglets; WP; weaners; FA: fatteners; r: correlation coefficient for the respective parameters with the SINS scores; P: significance of r; n.s.: not significant.

**Additional Table 4:** Red and white blood count according to SINS grades

| Parameter | Age  group | SINS low | SINS med | SINS high | r | P |
| --- | --- | --- | --- | --- | --- | --- |
| RBC | SP | 3.96 ± 0.79 | 3.71 ± 0.97 | 3.94 ± 0.79 | -0.010 | n.s. |
| (10^12^/L) | WP | 5.93 ± 1.23 | 6.11 ± 0.78 | 5.9 ± 0.84 | -0.026 | n.s. |
|  | FA | 8.37 ± 0.87 | 8.16 ± 1.67 | 7.77 ± 1.68 | -0.048 | n.s. |
| Hb | SP | 7.62 ± 1.27 | 7.45 ± 1.77 | 7.84 ± 1.27 | -0.001 | n.s. |
| (g/dL) | WP | 10.24 ± 2.23 | 10.54 ± 1.48 | 10.78 ± 1.33 | 0.076 | n.s. |
|  | FA | 14.46 ± 1.52 | 14.61 ± 3.21 | 16.55 ±0.71 | **0.260** | **0.008.** |
| HCT | SP | 23.92 ± 4.73 | 22.79 ± 5.93 | 24.02 ± 4.72 | 0.017 | n.s. |
| (%) | WP | 32.99 ± 7.37 | 33.02 ± 4.96 | 32.98 ± 4.7 | -0.069 | n.s. |
|  | FA | 43.86 ± 4.663 | 44.27 ± 9.45 | 47.20 ± 1.77 | **0.273** | **0.005** |
| MCV | SP | 60.57 ± 3.92 | 61.54 ± 3.99 | 61.08 ± 3.41 | 0.099 | n.s. |
| (fL) | WP | 55 ± 7.24 | 54.25 ± 5.93 | 56.09 ± 4.17 | -0.024 | n.s. |
|  | FA | 52.78 ± 3.94 | 53.6 ± 2.22 | 55.75 ± 2.87 | **0.377** | **<0.001** |
| MCH | SP | 19.5 ± 2.16 | 20.29 ± 2.35 | 20.16 ± 2.31 | 0.015 | n.s. |
| (pg) | WP | 17.17 ± 2.24 | 17.3 ± 2 | 18.46 ± 2.5 | 0.139 | n.s. |
|  | FA | 17.36 ± 1.2 | 17.78 ± 1.15 | 19.08 ± 1.36 | **0.326** | **0.001** |
| MCHC | SP | 32.22 ± 3.07 | 33.03 ± 3.62 | 33.02 ± 3.94 | -0.038 | n.s. |
| (g/dL) | WP | 31.27 ± 1.91 | 32.02 ± 2.09 | 32.95 ± 3.69 | **0.297** | **0.002** |
|  | FA | 32.94 ± 1.47 | 33.19 ± 1.31 | 34.28 ± 1.74 | -0.052 | n.s. |
| RDW | SP | 23.25 ± 2.03 | 22.29 ± 1.79 | 22.71 ± 1.52 | -0.059 | n.s. |
| (%) | WP | 24.22 ± 3.63 | 25.31 ± 5.1 | 23.79 ± 2.74 | -0.094 | n.s. |
|  | FA | 23.83 ± 1.19 | 22.92 ± 0.96 | 22.63 ± 0.94 | **-0.333** | **0.001** |

Hb: haemoglobin, HCT: haematocrit, LYM: lymphocytes, MCH: mean corpuscular haemoglobin, MCHC: mean corpuscular haemoglobin concentration, MCV: mean corpuscular volume. SP: suckling piglets; WP; weaners; FA: fatteners; r: correlation coefficient for the respective parameters with the SINS scores; P: significance of r; n.s.: not significant.

**Additional Table 5:** Serum electrolytes according to SINS grades

| Parameter | Age  group | SINS low | SINS med | SINS high | r | P |
| --- | --- | --- | --- | --- | --- | --- |
| Na | SP | 140.13 ± 4.68 | 140.69 ± 4.29 | 142.76 ± 5.22 | **0.200** | **.032** |
|  | WP | 133.83 ± 4.89 | 133.81 ± 4.76 | 135.13 ± 4.21 | 0.160 | n.s. |
|  | FA | 141 ± 5.07 | 140.8 ± 2.3 | 141.75 ± 3.86 | -0.07 | n.s. |
| Cl | SP | 102.74 ± 4.15 | 102.33 ± 4.29 | 102.92 ± 4.16 | 0.020 | n.s. |
|  | WP | 101.88 ± 6.24 | 102.75 ± 4.14 | 103.17 ± 4.59 | 0.144 | n.s. |
|  | FA | 97.51 ± 3.32 | 97.9 ± 1.85 | 100.5 ± 2.52 | -0.021 | n.s. |
| K | SP | 3.35 ± 0.35 | 3.43 ± 0.47 | 3.32 ± 0.38 | -0.005 | n.s. |
|  | WP | 3.55 ± 0.4 | 3.71 ± 0.65 | 3.83 ± 0.69 | 0.157 | n.s. |
|  | FA | 9.59 ± 1.68 | 10.39 ± 1.65 | 10.43 ± 1.58 | 0.121 | n.s. |
| Ca | SP | 2.8 ± 0.16 | 2.75 ± 0.23 | 2.71 ± 0.23 | **-0.189** | **0.043** |
|  | WP | 2.4 ± 0.17 | 2.37 ± 0.22 | 2.32 ± 0.16 | -0.179 | n.s. |
|  | FA | 2.83 ± 0.21 | 2.73 ± 0.08 | 2.85 ± 0.23 | -0.083 | n.s. |
| P | SP | 2.7 ± 0.56 | 2.55 ± 0.58 | 2.54 ± 0.72 | -0.072 | n.s. |
|  | WP | 2.15 ± 0.36 | 2.21 ± 0.32 | 2.23 ± 0.4 | -0.042 | n.s. |
|  | FA | 3.92 ± 0.42 | 4 ± 0.33 | 4.29 ± 0.28 | 0.087 | n.s. |
| Mg | SP | 0.78 ± 0.09 | 0.76 ± 0.12 | 0.77 ± 0.08 | 0.007 | n.s. |
|  | WP | 0.84 ± 0.08 | 0.84 ± 0.12 | 0.83 ± 0.12 | -0.102 | n.s. |
|  | FA | 1.19 ± 0.13 | 1.05 ± 0.09 | 1.17 ± 0.09 | -0.146 | n.s. |

SP: suckling piglets; WP; weaners; FA: fatteners; r: correlation coefficient for the respective parameters with the SINS scores; P: significance of r; n.s.: not significant.
